# Reviewing the Pathogenic Potential of the Otitis-Associated Bacteria *Alloiococcus otitidis* and *Turicella otitidis*

**DOI:** 10.3389/fcimb.2020.00051

**Published:** 2020-02-14

**Authors:** Rachael Lappan, Sarra E. Jamieson, Christopher S. Peacock

**Affiliations:** ^1^The Marshall Centre for Infectious Diseases Research and Training, School of Biomedical Sciences, The University of Western Australia, Perth, WA, Australia; ^2^Wesfarmers Centre of Vaccines and Infectious Diseases, Telethon Kids Institute, The University of Western Australia, Perth, WA, Australia; ^3^Telethon Kids Institute, The University of Western Australia, Perth, WA, Australia

**Keywords:** *Alloiococcus*, *Turicella*, otitis media, middle ear, otopathogen

## Abstract

*Alloiococcus otitidis* and *Turicella otitidis* are common bacteria of the human ear. They have frequently been isolated from the middle ear of children with otitis media (OM), though their potential role in this disease remains unclear and confounded due to their presence as commensal inhabitants of the external auditory canal. In this review, we summarize the current literature on these organisms with an emphasis on their role in OM. Much of the literature focuses on the presence and abundance of these organisms, and little work has been done to explore their activity in the middle ear. We find there is currently insufficient evidence available to determine whether these organisms are pathogens, commensals or contribute indirectly to the pathogenesis of OM. However, building on the knowledge currently available, we suggest future approaches aimed at providing stronger evidence to determine whether *A. otitidis* and *T. otitidis* are involved in the pathogenesis of OM. Such evidence will increase our understanding of the microbial risk factors contributing to OM and may lead to novel treatment approaches for severe and recurrent disease.

## Introduction

Otitis media (OM) is a polymicrobial disease most common in young children, characterized by inflammation of the middle ear and the presence of fluid behind the tympanic membrane. OM is a significant health care burden, affecting over 80% of children by the age of 3 (Teele et al., 1989) at an estimated annual cost of AUD$100 to 400 million in Australia (Taylor et al., 2009). The signs and symptoms of OM have been described as a phenotypic landscape of disease (Bhutta, 2014), but are commonly divided into two broad categories; acute OM (AOM) and OM with effusion (OME). In AOM, signs of an acute infection are present with a bulging tympanic membrane and often purulent fluid in the middle ear. Episodes of AOM may recur, becoming recurrent AOM (rAOM) if 3 or more episodes are experienced within 6 months, or ≥ 4 in 12 months (Kong and Coates, 2009). In OME, there are no signs of acute infection with the fluid more commonly serous or mucoid. If the fluid persists for ≥3 months, this is diagnosed as chronic OME (COME) (Bluestone et al., 2002). The onset of AOM will often occur subsequent to a viral upper respiratory infection, and may develop into OME (Bhutta, 2014). Chronic suppurative OM (CSOM) may follow untreated rAOM or COME, where the tympanic membrane perforates and there is persistent discharge (otorrhea) from the middle ear (Kong and Coates, 2009), allowing infection from external sources. Untreated OM can result in conductive hearing loss and consequent speech and language delays (Kong and Coates, 2009).

Three major bacterial pathogens are known to be involved in OM (known as otopathogens); *Streptococcus pneumoniae*, non-typeable *Haemophilus influenzae* (NTHi), and *Moraxella catarrhalis*. These otopathogens are frequently detected in middle ear fluid (MEF) from both children with OME and with AOM (Bluestone et al., 1992). As colonizers of the nasopharynx, these otopathogens are thought to ascend the Eustachian tube to the middle ear, often following an episode of an upper respiratory tract viral infection (Chonmaitree et al., 2008). Respiratory viruses are capable of increasing the adherence of otopathogens to host cells, compromising Eustachian tube function, stimulating the immune system and inducing mucus production which may contribute to the otopathogens' ability to colonize the middle ear (Bakaletz, 2002). These otopathogens are capable of intracellular invasion and the formation of biofilm in the middle ear (Thornton et al., 2011); a matrix of extracellular DNA, polysaccharides and proteins. With these mechanisms, otopathogens are protected from antibiotic molecules and the host immune system and are able to later recolonise the middle ear, likely contributing to the recurrent or persistent nature of severe OM (Hall-Stoodley et al., 2006). Colonization with multiple otopathogens in the nasopharynx is more common in children with rAOM than in healthy children (Wiertsema et al., 2011). However, in some studies utilizing PCR, substantial proportions (18–31%) of children with AOM do not appear to have any evidence of the three major otopathogens in the middle ear fluid (Leskinen et al., 2004; Harimaya et al., 2006a; Kaur et al., 2010; Holder et al., 2012); suggesting other microbes are also involved in the pathogenesis of OM.

There are other bacterial species often isolated from the middle ear fluid of children with OM that are not currently considered major otopathogens. The two organisms most frequently reported are *Alloiococcus otitidis* and *Turicella otitidis*. These bacteria have primarily been observed in the middle ear fluid of children with OME (Ashhurst-Smith et al., 2007; Jervis-Bardy et al., 2015; Chan et al., 2016), including Indigenous Australian children; however, these organisms have also been identified as normal flora of the external auditory canal (Stroman et al., 2001). It has been speculated for over 25 years that these species are involved in the development of OM (Faden and Dryja, 1989; Funke et al., 1994), but there remains no substantial evidence describing their role, if any, in the disease.

In this review, we aim to summarize the current knowledge on these two species and their association with OM. We extensively review reports of their prevalence and abundance in the MEF from children with OM by culture, species-specific PCR and next generation sequencing methods, which have limitations in accurately estimating the abundance of these organisms. We explore the major issues with interpreting these organisms as otopathogens, including their natural colonization of the external ear canal and their relative absence in the nasopharynx; features not characteristic of major otopathogen species. We also evaluate the minimal evidence available from studies that have aimed to demonstrate a pathogenic role. As these organisms' involvement in OM currently remains equivocal we propose directions for future research that could firmly establish how these “controversial” organisms relate to the health of children with OM.

## Alloiococcus otitidis

*A. otitidis* is a Gram positive coccus first reported as an unknown organism isolated from the MEF of children with OME in 1989 (Faden and Dryja, 1989). It was characterized as slow-growing, aerobic, catalase positive, and oxidase negative and was distinguishable from the phenotypically similar *Aerococcus, Gemella, Enterococcus*, and *Micrococcus* (Faden and Dryja, 1989). In 1992, the 16S rRNA sequence of the novel organism was analyzed and it was named *Alloiococcus* (“different coccus”) *otitis* (Aguirre and Collins, 1992). This nomenclature was later revised (von Graevenitz, 1993) to *A. otitidis*. Placed within the *Carnobacteriaceae* family, it is the only species in its genus and is most closely related to *Dolosigranulum* by 16S rRNA sequence homology (Vos et al., 2009). *Dolosigranulum* is a nasopharyngeal commensal of relevance to OM, and their similarity leads to misclassification in 16S rRNA gene surveys using older taxonomic databases (Lappan et al., 2018). *A. otitidis* strains have been characterized as resistant to trimethoprim-sulfamethoxazole and macrolides, and susceptible or intermediately resistant to penicillin and ampicillin (Bosley et al., 1995; Ashhurst-Smith et al., 2007; Marsh et al., 2012), though they are β-lactamase negative (Bosley et al., 1995).

## Turicella otitidis

The first description of *T. otitidis* was in 1993, when it was independently determined that unidentified coryneforms from MEF specimens (primarily from children with AOM), whilst phenotypically similar to *Corynebacterium afermentans*, were biochemically distinct from this species (Funke et al., 1993; Simonet et al., 1993). The 16S rRNA sequences of these strains showed they were also phylogenetically distinct and the new genus and species *T. otitidis* was proposed (Funke et al., 1994). *T. otitidis* is a Gram positive bacillus; catalase positive, oxidase negative and aerobic (Funke et al., 1994). Like *A. otitidis, T. otitidis* is the only member of its genus and was placed in the *Corynebacteriaceae* family with its closest relative, *Corynebacterium* (Goodfellow et al., 2012); though it has recently been proposed that *T. otitidis* is a true member of the *Corynebacterium* genus (Baek et al., 2018). Like *A. otitidis, T. otitidis'* closest relative is also a nasopharyngeal commensal, and *Corynebacterium* and *Dolosigranulum* commonly co-occur in healthy children (Biesbroek et al., 2014; Lappan et al., 2018). *T. otitidis* is similarly misclassified as *Corynebacterium* in at least one ribosomal taxonomic database (Lappan et al., 2018). *T. otitidis*is sensitive to tetracyclines and amoxicillin, but is resistant to sulfamethoxazole and co-trimoxazole (Troxler et al., 2001) and some strains have developed mutations conferring macrolide resistance (Gómez-Garcés et al., 2004; Boumghar-Bourtchai et al., 2009).

## Why Have These Organisms Been Implicated in Otitis Media?

Both *Alloiococcus* and *Turicella* were first isolated by culture from the MEF of children with OM, prompting suggestions of their association with the disease (Faden and Dryja, 1989; Funke et al., 1993; Simonet et al., 1993). This is the primary niche from which these organisms have since been consistently detected, though they have not been studied as extensively as the three major otopathogen species. *A. otitidis* has appeared more frequently than *T. otitidis* in the scientific literature, with 97 results for “*Alloiococcus”* and 44 for “*Turicella”* in the Scopus database as at 2019-02-07; this literature has been used for this review. This disparity is likely because *A. otitidis* can be detected by both culture and PCR methods, whereas there are currently no published primer pairs specific to *T. otitidis*. Additionally, cultured *T. otitidis* isolates are not often distinguished from commensal skin coryneforms (von Graevenitz and Funke, 2014). Table 1 summarizes the studies that have evaluated the prevalence of *A. otitidis* in MEF by either microbiological culture or targeted PCR, including the OM phenotype and extent of ear canal avoidance for each. Few studies describe the prevalence of *T. otitidis* (see section *T. otitidis* Has Been Detected in MEF by Culture, But Is Seldom Reported).

**Table 1:** Detection of *A. otitidis* in MEF specimens by culture and PCR methods.

**References**	**Phenotype**	**Method of detection**	***A. otitidis* prevalence**	**Major otopathogen prevalence**^****a****^			**Contact with the ear canal**	**Participants**	**Culture details for *A. otitidis***
				**Hi**	**Spn**	**Mcat**			
Faden and Dryja (1989)	OME	Culture	16 isolates of *A. otitidis* from 10 children	23%	14%	11%	Not described	320 samples from 200 children aged 0–2 years	37°C with 5% CO_2_ for 2–5 days on blood agar
Hendolin et al. (1997)	OME (effusion ≥ 1 month)	Culture PCR	Not cultured 5/25 (20%)	2/25 (8%) 13/25 (52%)	2/25 (8%) 2/25 (8%)	4/25 (16%) 4/25 (16%)	Ear canal mechanically cleaned	25 samples from 16 children aged 1–7 years (median age 3 years)	NA
Beswick et al. (1999)	COME (effusion ≥ 6 months)	Culture PCR^b^	0/12 (0%) 6/12 (50%)	0/12 (0%) 1/12 (8.3%)	0/12 (0%) 0/12 (0%)	0/12 (0%) 1/12 (8.3%)	Care taken to avoid canal via sealed sampling system	12 samples from 10 children aged 5–10 years and 2 adults (28 and 59 y)	37°C with 5% CO_2_ for a maximum of 5 days on blood agar
Hendolin et al. (1999)	OME	Culture PCR	0/67 (0%) 31/67 (46.3%)	6/67 (9%) 12/67 (17.9%)	2/67 (3%) 14/67 (20.9%)	6/67 (9%) 25/67 (37.3%)	Ear canal mechanically cleaned, all children had intact tympanic membrane	67 samples from 48 children aged 13 months−9 years and 2 months (median age 3 years 8 months)	Conditions not specified, on blood agar
Hendolin et al. (2000)	OME	PCR	14/73 (19.2%)	24/73 (32.9%)	26/73 (35.6%)	39/73 (53.4%)	Ear canal mechanically cleaned	73 samples from children	NA
Kalcioglu et al. (2002)^c^	COME	PCR	10/54 (18.5%)	7/54 (13%)	2/54 (3.7%)	4/54 (7.4%)	Not available	54 samples from 32 children	NA
Leskinen et al. (2002)	OME (effusion ≥ 1 month)	Culture PCR	0/123 (0%) 25/123 (20.3%)	18/123 (14.6%) 40/123 (32.5%)	14/123 (11.4%) 43/123 (35%)	8/123 (6.5%) 78/123 (63.4%)	Cleaned of cerumen, all children had intact tympanic membranes	123 samples from 123 children aged 7 months−12 years (median age 2 years 5 months)	Not described
Leskinen et al. (2004)	AOM	Culture PCR	NA 30/118 (25.4%)	22/118 (18.6%) 13/118 (11%)	26/118 (22%) 24/118 (20.3%)	12/118 (10.2%) 32/118 (27.1%)	Excluded children with spontaneous perforations or current tubes. 10% had previously had tympanostomy.	118 samples from 118 children aged 3 months−7 years 5 months (median age 2 years 6 months)	NA
Pereira et al. (2004)^d^	rAOM and COME (effusion ≥ 3 months)	Culture PCR	0/128 (0%) 67/128 (52.3%)	13/128 (10.2%) 50/128 (39.1%)	8/128 (6.3%) 16/128 (12.5%)	5/128 (3.9%) 13/128 (10.2%)	Cerumen removed and canal disinfected with 70% alcohol	128 samples from 75 children aged 11 months−10 years	37°C for a maximum of 5 days on blood and chocolate agar
Harimaya et al. (2006a)	AOM and COME (effusion ≥ 3 months)	Culture	0/40 (0%) in AOM 0/76 (0%) in OME	2/40 (5%) in AOM 4/76 (5.3%) in OME	5/40 (12.5%) in AOM 1/76 (1.3%) in OME	0/40 (0%) in AOM 1/76 (1.3%) in OME	Disinfected with povidone-iodine	116 samples from 88 children aged 9 months to 8 years (median age 3.5 years) for AOM; 6 months to 12 years (median age 4 years) for OME.	Conditions not specified, for up to 14 days on blood and chocolate agar
		PCR	20/40 (50%) in AOM 46/76 (60.5%) in OME	3/40 (7.5%) in AOM 9/76 (11/8%) in OME	5/40 (12.5%) in AOM 6/76 (7.9%) in OME	8/40 (20%) in AOM 5/76 (6.6%) in OME			
Harimaya et al. (2006b)	recurrent OM (“otitis-prone”) and non-recurrent OM (“non-otitis-prone”)	Culture PCR	0/83 (0%) 16/25 (64%) in recurrent OM 8/58 (13.8%) in non-recurrent OM	NA	NA	NA	Disinfected with povidone-iodine	83 samples from 56 children aged 8 months to 10 years (median age 4 years)	Conditions not specified, for up to 14 days on blood and chocolate agar
Ashhurst-Smith et al. (2007)	COME	Culture	20/50 (40%)	2/50 (4%)	1/50 (2%)	0/50 (0%)	Cerumen removed and canal disinfected with povidone-iodine	50 samples from 50 children aged 1–10 years	35°C in 7.5% CO_2_ for 7 days on blood, chocolate, MacConkey and colistin-nalidixic acid blood agar
de Miguel Martínez and Ramos Macías (2008)	AOM and OME	Culture	0/40 (0%) in AOM 14/40 (35%) in OME	10/40 (25%) in AOM 5/40 (12.5%) in OME	16/40 (40%) in AOM 1/40 (2.5%) in OME	0/80 (0%)	External ear canal was avoided, and children with previous tubes or perforations excluded	80 samples from 80 children with OME (mean age 4.1 years) and AOM (mean age 3.2 years)	37°C for 3 days on blood agar
Güvenç et al. (2010)	OME	PCR^b^	12/46 (26.1%)	7/46 (15.2%)	1/46 (2.2%)	1/46 (2.2%)	Disinfected with 70% alcohol and care taken to avoid contact during sampling	46 samples from 28 children aged 2–12 years (mean age 7 years)	NA
Kaur et al. (2010)	AOM	Culture PCR (culture-negative samples)	0/170 (0%) 16/49 (32.7%)	54/170 (31.8%) 17/49 (34.7%)	35/170 (20.6%) 25/49 (51%)	13/170 (7.6%) 7/49 (14.3%)	Not described	170 samples from 97 children aged 6 months−3 years	37°C with 5% CO_2_, time not specified, on blood and chocolate agar^e^
Aydin et al. (2012)	COME (effusion ≥ 3 months)	Culture PCR	0/34 (0%) 12/34 (35.3%)	0/34 (0%) 1/34 (2.9%)	0/34 (0%) 3/34 (8.8%)	0/34 (0%) 3/34 (8.8%)	Disinfected with povidone-iodine	34 samples from 34 children aged 3 16 years (mean age 8 years)	35°C with 5% CO_2_ for 7 days on blood and chocolate agar
Holder et al. (2012)	rAOM and COME	PCR	7/38 (18.4%) in purulent MEF 43/169 (25.4%) in non-purulent MEF	25/38 (65.8%) in purulent MEF 40/169 (23.7%) in non-purulent MEF	2/38 (5.3%) in purulent MEF 8/169 (4.7%) in non-purulent MEF	7/38 (18.4%) in purulent MEF 29/169 (17.2%) in non-purulent MEF	Not described. Children with previous ear tubes included.	207 samples from 207 children aged < 18 years (50% aged 1–3 years)	NA
Khoramrooz et al. (2012)	COME (effusion ≥ 3 months)	Culture PCR	15/63 (23.8%) 25/63 (39.7%)	3/63 (4.8%) 7/63 (11.1%)	6/63 (9.5%) 7/63 (11.1%)	6/63 (9.5%) 6/63 (9.5%)	Disinfected with povidone-iodine and children with perforations or previous tubes excluded	63 samples from 48 children aged 1.7–12 years (mean age 7 years)	35°C in 5% CO_2_ for 14 days on blood agar
Marsh et al. (2012)	AOM with perforation	Culture qPCR	5/11 (45.5%) of qPCR-positive swabs 11/31 (35.4%)	4/11 Ao-positive swabs 10/11 Ao-positive swabs	2/11 Ao-positive swabs 3/11 Ao-positive swabs	1/11 Ao-positive swabs 4/11 Ao-positive swabs	Samples were ear discharge swabs from perforations	31 samples from 27 Indigenous Australian children aged 6 months−4 years (median age 1.2 years)	37°C for 2–21 days on blood agar
Garibpour et al. (2013)^d^	OME	Culture PCR	15/65 (23%) 26/65 (40%)	NA	NA	NA	Not available	65 samples from 50 children	Not available
Holder et al. (2015)	AOM and OME (purulent and non-purulent MEF)	PCR	7/31 (22.6%) purulent MEF 63/245 (25.7%) non purulent MEF	16/31 (51.6%) in purulent MEF 45/245 (18.4%) in non-purulent MEF	6/31 (19.4%) in purulent MEF 9/245 (3.7%) in non-purulent MEF	8/31 (25.8%) purulent MEF 30/245 (12.2%) nonpurulent MEF	Not described. Children with previous tubes included.	276 samples from 276 children aged 0–18 years (mean age 2.7 years)	NA
Sheikh et al. (2015)	OME	Culture PCR	1/70 (1.4%) 18/70 (25.7%)	0/70 (0%) 14/70 (20%)	2/70 (2.8%) 14/70 (20%)	3/70 (4.2%) 9/70 (12.9%)	Disinfected with 70% alcohol, MEF collected by swab	70 samples from 45 children aged 1–15 years (mean 4.5 years)	35°C with 5% CO_2_ for 2 weeks on blood agar
Slinger et al. (2016)	OME	Culture qPCR	0/48 (0%) 15/48 (31.3%)	3/48 (6.3%) 5/48 (10.4%)	2/48 (4.2%) 15/48 (31.3%)	5/48 (10.4%) 14/48 (29.2%)	Disinfected with 70% alcohol	48 samples from 30 children aged 11 months−10 years (median age 2.8 years)	37°C for 5 days on blood, chocolate and MacConkey agar
Sillanpää et al. (2016)	AOM (some with perforation)	Culture qPCR	0/90 (0%) 6/90 (6.7%)	17/90 (18.9%) 30/90 (33.3%)	15/90 (16.7%) 27/90 (30%)	8/90 (8.9%) 42/90 (46.7%)	Some children had drainage from tube or perforation	90 samples from 79 children aged 5 months−3.3 years (median age 1.6 years)	35°C with 5% CO_2_ for 24 hours on blood and chocolate agar

PCR detection utilized species-specific primers unless otherwise indicated.

a Hi, Haemophilus influenzae; Spn, Streptococcus pneumoniae; Mcat, Moraxella catarrhalis; Ao, Alloiococcus otitidis.

b PCR targeting 16S rRNA, amplicons identified by sequencing.

c Only abstract available.

d Only abstract and tables available in English.

e *Casey et al. (2010)*.

### *A. otitidis* Can Be Difficult to Detect by Culture

*A. otitidis* was originally detected by microbiological culture from MEF and was thus implicated as a cause of OME. However, it has remained a challenging organism to grow, requiring extended incubation times and producing small colonies (Ashhurst-Smith et al., 2007) so it may have been overlooked as an otopathogen in early studies. At its discovery, it was successfully cultured at 37°C with 5% CO_2_ on blood agar for 2–5 days (Faden and Dryja, 1989). Subsequent studies apparently failed to culture the organism, with rates of 0% reported until 2007 when Ashhurst-Smith et al. (2007) successfully cultured the organism in 40% of MEF samples using a slightly lower temperature (35°C) and higher CO_2_ (7.5%). Only six studies since the 1989 paper have reported the prevalence of *A. otitidis* in MEF by culture; five cultured it from 1 to 40% of samples from children with OME or COME (Ashhurst-Smith et al., 2007; de Miguel Martínez and Ramos Macías, 2008; Khoramrooz et al., 2012; Garibpour et al., 2013; Sheikh et al., 2015) and one cultured it from 46% of qPCR-positive otorrhoea swabs from children with AOM and perforation (Marsh et al., 2012). Due to the varying culture conditions used to isolate the organism (see Table 1) and the potential for overgrowth of other organisms to mask the presence of small *A. otitidis* colonies, it is not clear whether the prevalence of *A. otitidis* by culture is comparable to that of the three major otopathogens. A clearer picture can be obtained from those studies that have detected these organisms via species-specific PCR.

### *A. otitidis* Is Commonly Detected in MEF by PCR

In 1997, *A. otitidis* was incorporated as part of a multiplex PCR method with the three major otopathogens, NTHi, *S. pneumonia*, and *M. catarrhalis* (Hendolin et al., 1997). The use of PCR has substantially improved the detection rate of these four organisms in MEF specimens compared to microbiological culture, as demonstrated by studies where both methods were used (Hendolin et al., 1997, 1999; Leskinen et al., 2002; Pereira et al., 2004; Harimaya et al., 2006a; Kaur et al., 2010; Aydin et al., 2012; Khoramrooz et al., 2012; Sheikh et al., 2015; Sillanpää et al., 2016; Slinger et al., 2016). In children with OME or COME, *A. otitidis* has been reported by PCR in 18.5–60.5% of MEF specimens (13 studies), and in children with AOM or rAOM, 7–50% (5 studies). Two studies compared the prevalence of *A. otitidis* in purulent and non-purulent MEF; *A. otitidis* was detected in 18.4 and 22.6% of purulent fluids, and in 25.4 and 25.7% of non-purulent fluids (Holder et al., 2012, 2015). However, there were 4–8 times as many non-purulent fluids tested in these studies as purulent fluids. One study has measured the bacterial load of *A. otitidis* in children with AOM with perforation and demonstrated that it was present at a comparable load to *H. influenzae*, the most dominant otopathogen (Marsh et al., 2012); but this is the only study to have done so.

The information currently available on the prevalence of *A. otitidis* suggests it is more commonly associated with persistence (OME) than acute infection (AOM). The within study-comparisons of purulent and non-purulent fluid support this (Holder et al., 2012, 2015), though no other studies have made a direct comparison between phenotypes and the difference in proportions is not large. It therefore may be hypothesized that the role of *A. otitidis* in OM is in the perpetuation of inflammation rather than as a direct pathogenic cause of acute infection. Further studies are needed to test this hypothesis, however some caution is required as the apparent association may be due to publication bias with few reported studies having sought to detect *A. otitidis* in children with AOM. Importantly, the prevalence and abundance of *A. otitidis* in MEF is possibly overestimated due to its habitation of the external ear canal. Several of the studies in Table 1 either did not describe measures that were taken to avoid contamination with the ear canal, disinfected it only with 70% alcohol (which does not destroy DNA) or included children whose tympanic membranes had previously been breached.

Of the 16 studies summarized in Table 1 that have reported the prevalence *A. otitidis, S. pneumoniae*, NTHi, and *M. catarrhalis* by species-specific PCR, *A. otitidis* was detected more frequently than each of the major otopathogens in 9 of them (for two of these studies, in non-purulent fluid only). Table 2 describes those studies that have reported whether *A. otitidis* occurs alone or is detected together with the major otopathogens. Both scenarios are reported, with *A. otitidis*, present in the fluid-filled middle ear with and without the presence of the major otopathogen species. Therefore, if *A. otitidis* is involved in the pathogenesis of OM, it may directly cause disease or it may contribute by supporting the main otopathogens in some way.

**Table 2:** Distribution of *A. otitidis* detected with at least one otopathogen species or as the sole detected organism.

**References**	**Detection with at least one otopathogen (%)**	**Detection as** **sole organism (%)**	**Phenotype**
Hendolin et al. (1999)	58	42	OME
Leskinen et al. (2002)	96	4	OME
Pereira et al. (2004)	45	55	rAOM and COME
Aydin et al. (2012)	42	58	COME
Holder et al. (2012)	71	29	Purulent fluid
	49	51	Non-purulent fluid
Holder et al. (2015)	71	29	Purulent fluid
	27	73	Non-purulent fluid

*Studies that have reported the number of A. otitidis-positive samples that also contained one or more otopathogenic species are included. All detection is by PCR. Percentages represent the proportion of total A. otitidis-positive samples*.

### *T. otitidis* Has Been Detected in MEF by Culture, but Is Seldom Reported

Detection of *T. otitidis* has been described infrequently in the literature. This is likely due to the difficulty in phenotypically distinguishing it from other coryneforms, which are often considered contaminants and do not appear to be routinely identified to species level (von Graevenitz and Funke, 2014). Additionally, no PCR primers have been described in the literature for the detection of *T. otitidis*, so it has only been detected by standard culture methods and next generation sequencing. Like *A. otitidis*, it has primarily been observed and reported in the MEF of children with OM, so both organisms may have been overlooked as otopathogens.

Since original detection of the organism in MEF in two independent studies (Funke et al., 1993; Simonet et al., 1993), very few studies have reported detection of *T. otitidis* by culture. Renaud et al. (1996) reported that otorrheal fluid from a child with a perforated tympanic membrane produced several colonies of *T. otitidis* (Renaud et al., 1996). Its prevalence appears to be low, with 7/112 (6.3%) of MEF samples in a 2004 study producing *T. otitidis* colonies (Gómez-Garcés et al., 2004). In 5 of these 7 samples the children had perforations and the MEF was collected from the external ear canal. One study has assessed the prevalence of *T. otitidis* where perforations were not present, reporting it in 10% of MEF samples from children with COME, compared to the major otopathogens at 3.3–8.3% (Holzmann et al., 2002). In this study, where *T. otitidis* was detected in the MEF, it was also detected in the external auditory canal; though the canal was disinfected between sampling each site. As we describe later in this review, the contribution of canal flora is challenging to tease out from the detection of both *A. otitidis* and *T. otitidis* in the MEF.

### Detection of *A. otitidis* and *T. otitidis* in MEF Has Increased With Next Generation Sequencing

In recent years, several studies have utilized next generation DNA sequencing to study the whole bacterial community present in MEF specimens (Table 3). By amplifying and sequencing a segment of the 16S rRNA gene, the microbial profile (or microbiome) can be characterized, including bacteria that are challenging to isolate in culture or are not targeted by commonly-used species-specific PCRs. The advantage of this method is that these potentially overlooked organisms can not only be detected, but their relative abundance in a sample can be estimated and compared between samples. However, the major disadvantage is that only part of the gene is sequenced, providing little to no taxonomic resolution beyond the genus level (or broader, depending on the taxa and the region sequenced).

**Table 3:** Genus-level relative abundance of *A. otitidis* and *T. otitidis* in MEF specimens by 16S rRNA gene sequencing.

**References**	**Phenotype**	***A. otitidis* relative abundance**	***T. otitidis* relative abundance**	**Main otopathogen relative abundance**			**Contact with the ear canal**	**Participants**
				***Haemophilus***	***Streptococcus***	***Moraxella***		
Jervis-Bardy et al. (2015)	COME (effusion ≥ 6 months)	>50% in 6/11 samples	Detected in 3/11 samples	>50% in 3/11 samples	>50% in 1/11 samples	Detected in 2/11 samples	Minimized contact	11 samples from Indigenous Australian children aged 3–9 years (mean age 5.3 years)
Chan et al. (2016)	COME (effusion > 3 months)	23% (cumulative)	Not reported	22% (cumulative)	5% (cumulative)	5% (cumulative)	Care taken to avoid contact	35 samples from 23 children aged 1–8 years (mean age 3.3 years)
Neeff et al. (2016)^a^	CSOM (with and without cholesteatoma) and healthy ME controls	Absent from healthy controls and non-cholesteatoma CSOM. ~10% in CSOM patients with cholesteatoma.	Not reported	Very low abundance in healthy controls. Absent from non-cholesteatoma CSOM. ~25% in CSOM patients with cholesteatoma.	Reported in both MEF and healthy ME, abundance not described.	Absent from healthy controls and cholesteatoma CSOM. < 1% in non-cholesteatoma CSOM.	All CSOM patients had perforations	22 samples from healthy controls aged 6 months−85 years, and 24 samples from CSOM patients aged 1–75 years (16 with and 8 without cholesteatoma).
Santos-Cortez et al. (2016)	CSOM	0% (median)	Not reported	0.17% (median)	Not reported	Not reported	All patients perforated. Canal also sampled.	16 Indigenous Filipino individuals aged 4–24 years (median age 9.5 years)
Chan et al. (2017b)	OME	37.5% (mean)	Not reported^b^	14.4% (mean)	3.8% (mean)	10.0% (mean)	Care taken to avoid direct contact. Canal also sampled. 18% had previous tubes.	18 samples from children aged 1–14 years (mean age 4 years)
Krueger et al. (2017)	COME	5.1%	7.8%	22.5%	4.2%	11.1%	Not described	55 samples from children aged 3 months−14.7 years (mean age 3.4 years)
Minami et al. (2017)^c^	COME and healthy ME controls	Not described	2% across all samples	Not described	Reported in one COME sample	Not described	Some patients with perforation. Care taken to avoid contact	88 individuals with COME (44 with active inflammation and MEF aged 9–84 years, mean age 57 years; 44 without active inflammation or MEF aged 6–83 years, mean age 59 years) and 67 healthy ME controls aged 1–72 years (mean age 22 years)
Sillanpää et al. (2017)^d^	AOM	0.7% (mean)	1.9% (mean)	14.0% (mean)	13.8% (mean)	6.1% (mean)	Some patients with perforation or previous tubes	90 samples from 79 children aged 5 months−3.5 years (median age 1.6 years)
Boers et al. (2018)^d^	rAOM and OME	24.9% (mean)	23.3% (mean)	9.1% (mean)	6.9% (mean)	0.26% (mean)	Some patients with previous tubes	19 samples from children aged 0.8–12.8 years
Lappan et al. (2018)	rAOM	49.8% (cumulative)	6.7% (cumulative)	18.5% (cumulative)	3.5% (cumulative)	2.2% (cumulative)	Canal also sampled	127 samples from children with median age 1.9 years (IQR 1.3–2.8y)
Val et al. (2018)^e^	COME (effusion > 3 months)	~15% (mean)	~6% (mean)	~20% (mean)	~6% (mean)	~12% (mean)	Not described	5 samples; mean age for this subset of cohort not reported.
Johnston et al. (2019)^f^	COME (effusion ≥ 6 months)	~12%	~7%	~11%	~6%	0%	Not described. MEF collected by swab.	10 samples from children aged 2–10 years (mean age 5 years)

Where the information was available, relative abundance is reported as either the total relative abundance across all sequence reads (cumulative) or mean/median relative abundance across all samples (mean/median). ME, middle ear.

a Values interpreted from Figure 3 (relative abundance of dominant genera).

b Possibly reported as Corynebacterium (3.1% mean relative abundance), as GreenGenes database was used for classification. The GreenGenes v13_8 database contains Turicella sequence labeled as its close relative Corynebacterium.

c This study primarily reported results at phylum level or for small groups of samples.

d Values calculated from Figure 1 (heatmap of relative abundance per sample with values).

e Values interpreted from Figure 5 (relative abundance of genera above 5%, per sample). If genus not shown, value taken as 0%.

f *Values estimated from Figure 1 (stacked barplot of genera across all MEF samples)*.

In the microbiota of MEF, *Alloiococcus* appears to be one of the dominant genera (Jervis-Bardy et al., 2015; Chan et al., 2016, 2017b; Boers et al., 2018; Lappan et al., 2018; Val et al., 2018; Johnston et al., 2019). *Turicella* is less often reported, but has been identified as a member of the MEF microbiota in 8 of the 12 MEF microbiota studies reported in Table 3 (Jervis-Bardy et al., 2015; Krueger et al., 2017; Minami et al., 2017; Sillanpää et al., 2017; Boers et al., 2018; Lappan et al., 2018; Val et al., 2018; Johnston et al., 2019). However, due to the compositional nature of microbiome data it remains difficult to interpret which organisms are more abundant than others. Microbiome composition may also vary with the efficacy of the chosen DNA extraction method and amplicon primers on different organisms, and the variation in numbers of copies of the 16S rRNA gene amongst different bacterial genera; qPCR is still a more reliable indicator of whether there are greater numbers of *Alloiococcus* than other organisms within the same sample.

### What Does Their Presence in MEF Tell us?

*A. otitidis*, and to a lesser extent *T. otitidis*, are prevalent in MEF specimens from children with OM. *A. otitidis* in particular is often detected at a similar or higher frequency to the three major otopathogen species. These two organisms may have been overlooked as potential otopathogens as they can be challenging to detect, though studies of the microbiome of MEF have revealed that they are not insignificant members of the microbiota of the otitis-prone middle ear. However, the observations from the studies summarized thus far only describe their prevalence and abundance, and do not assess their potential role in the pathogenesis of OM.

## Why Is the Role of These Organisms in Otitis Media Still Under Debate?

Despite their prevalence in the MEF of children with OM, the role of *A. otitidis* and *T. otitidis* as potential otopathogens remains under debate. This is mostly due to two complicating factors: their presence in the external auditory canal (EAC), where the major otopathogens are rarely observed; and their infrequency in the nasopharynx, the site where major otopathogens initially colonize before ascension via the Eustachian tube to the middle ear.

### *A. otitidis* and *T. otitidis* Are Found in the External Auditory Canal

When first discovered, *A. otitidis* was reported not to be found in the EAC (Faden and Dryja, 1989), but both *A. otitidis* and *T. otitidis* have since been proposed as members of the normal EAC flora. Both organisms have been observed in the EACs of healthy adults and children (Stroman et al., 2001; Frank et al., 2003; Tano et al., 2008; De Baere et al., 2010), indicating that they are capable of growth in the EAC without causing disease. Given this finding, it becomes important to establish whether the detection of *A. otitidis* and *T. otitidis* in MEF is a result of contamination during sampling, or if they inhabit both sites. Currently, this is difficult to determine as only three studies, to our knowledge, have been in a position to address this question by looking for these organisms in EAC and MEF samples from the same ear in children with OM and intact tympanic membranes.

A 2002 study cultured *T. otitidis* from the EAC and MEF of children with OME, and found that the frequency of *T. otitidis* was higher in the EAC of children with OME (23%) than in healthy control children (11%) (Holzmann et al., 2002). Additionally, they noted that it was never isolated from the MEF alone; every MEF in which it was present had a positive result for the corresponding EAC sample. Because of this finding, they suggested that it resides only in the EAC, noting that many studies observing *T. otitidis* in MEF did not look for it in the EAC and that this was the likely source.

A recent study by Chan et al. (2017b) using 16S rRNA amplicon sequencing on samples from the EAC and MEF of children with OME did not observe *T. otitidis*, but reported *A. otitidis* as the most abundant species in both sites. They noted its high abundance in children who had previously had grommets, and suggested that the EAC may serve as a reservoir of infection for the middle ear via a tympanic membrane perforation. This study detailed their care in sampling the MEF without touching the EAC and concluded it unlikely that the abundance of *A. otitidis* in the MEF was the result of contamination from the EAC during sampling.

We recently took a similar approach, undertaking 16S rRNA amplicon sequencing on EAC and MEF samples from children with rAOM (Lappan et al., 2018). We observed *A. otitidis* and *T. otitidis* as dominant organisms in both sites in a cohort where the majority of children had no known current tympanic membrane perforations or previous grommets. While it is unlikely that these children had suffered tympanic membrane perforations, a perforation history was not obtained. The relative abundance of both *A. otitidis* and *T. otitidis* was significantly higher in the EAC than in the MEF. As with the Chan et al. study, care was taken to ensure the MEF was sampled without touching the EAC, however as we did not identify any bacteria unique to the EAC we could not rule out contamination of the MEF during sampling.

Based on the studies above, it is plausible that these organisms are present in both sites, but the evidence currently suggests that their primary habitat is the EAC. In support of this possibility, other studies have detected *A. otitidis* and *T. otitidis* in the EAC by culture and PCR, commonly observing a higher frequency in the EAC of children with OM than in healthy children (Gómez-Hernando et al., 1999; Durmaz et al., 2002; Holzmann et al., 2002). Tano et al. found *A. otitidis* more frequently in the EAC of healthy individuals (29%) than patients with OM (6%), but sampled from adults and children and did not report these results separately (Tano et al., 2008). In the EAC of healthy children and adults, Stroman et al. (2001) cultured *A. otitidis* as the most common streptococci/enterococci-like organism, and *T. otitidis* as the dominant coryneform. While most of the *A. otitidis* isolates were from children, it was also detected in adults and *T. otitidis* isolates were found across both age groups. Frank et al. (2003) undertook a similar assessment of EAC samples from adults and children using 16S rRNA cloning and sequencing. They observed *A. otitidis* and *T. otitidis* (referred to as *Corynebacterium otitidis*) as the most prevalent sequence types, representing 57 and 20% of all clones, respectively, and with *A. otitidis* common in children. A more recent study also suggested that the organisms are not specific to children, detecting *A. otitidis* by PCR in 83% of EAC samples from healthy young adults, where *T. otitidis* was also cultured from 5 of 10 randomly selected samples (De Baere et al., 2010).

It therefore seems likely that *A. otitidis* and *T. otitidis* found in MEF originate from the EAC; but if these organisms do colonize the middle ear, their mechanism of entry remains unknown. It has been hypothesized that these organisms could enter the middle ear through a perforation in the tympanic membrane (De Baere et al., 2010; Marsh et al., 2012; Chan et al., 2017b). However, there are some studies that have reported the detection of *A. otitidis* in children with intact tympanic membranes when care is taken to avoid contact with the EAC (Leskinen et al., 2002, 2004; de Miguel Martínez and Ramos Macías, 2008; Khoramrooz et al., 2012). It is possible that small perforations sufficient for bacterial entry may not be detected by parents or physicians; and perhaps inflammation of the middle ear increases permeability of the tympanic membrane. However, understanding their entry into the middle ear does not indicate whether they are responsible for causing disease once inside. The sparsity of the major otopathogens in the EAC compared to *A. otitidis* and *T. otitidis* (Stroman et al., 2001; Frank et al., 2003; De Baere et al., 2010; Chan et al., 2017b; Lappan et al., 2018) indicates that *A. otitidis* and *T. otitidis* either have a different pathway for involvement in OM compared to the major otopathogens, or they are not otopathogenic organisms.

### They Are Infrequently Found in the Nasopharynx

*A. otitidis* and *T. otitidis* show dissimilarity to the major otopathogens not only because they are found in the EAC, but also because they are rarely found in the nasopharynx. It is commonly accepted that the major otopathogens colonize the nasopharynx before ascension to the middle ear and subsequent infection (Chonmaitree et al., 2008). The low detection rates of *A. otitidis* and *T. otitidis* in the nasopharynx are another indicator that these organisms behave differently compared to the major otopathogens.

*A. otitidis* has infrequently been reported in the nasopharynx, and then only by PCR. The first study to do so was Durmaz et al. (2002), where it was detected in 15% of 27 nasopharyngeal samples from children with chronic OM (Durmaz et al., 2002). It has been observed more frequently in the nasopharynx of children with a history of otitis media episodes (>3 in 6 months, >4 in 12 months, or > 4 by 2 years of age; 29.4%) than those with fewer episodes (2.6%) (Harimaya et al., 2006b). It was also observed in 9% of nasopharyngeal samples from children with AOM (Kaur et al., 2010) and in 7% of children with an upper respiratory tract infection (Tano et al., 2008). It is very rare in the nasopharynx of healthy children, seen in 0.5% in one study (Janapatla et al., 2011) whilst in others was not detected (Durmaz et al., 2002; Aydin et al., 2012). It was also found to be absent from the nasopharynx of healthy young adults (De Baere et al., 2010). Where these studies also observed *A. otitidis* in the MEF or ear canal, it was found more commonly in these sites than in the nasopharynx (Harimaya et al., 2006b; Tano et al., 2008; De Baere et al., 2010; Kaur et al., 2010; Aydin et al., 2012), with one exception (Durmaz et al., 2002). *A. otitidis* also appears to be absent, or has been detected at very low abundance, on the tonsils (Aydin et al., 2012) and adenoids (Khoramrooz et al., 2012; Jervis-Bardy et al., 2015; Chan et al., 2016) of children with OME, suggesting that these lymphatic tissues are not a source of the organism in the middle ear. To our knowledge, *T. otitidis* has not been reported in the nasopharynx by culture or by PCR.

Both *A. otitidis* and *T. otitidis* have occasionally been detected in the nasopharynx by 16S rRNA amplicon sequencing. However, both organisms are likely to be misclassified depending on the reference database used. *A. otitidis* was reported in the nasopharynx of healthy infants (Teo et al., 2015) and in sinonasal swabs from adults with chronic rhinosinusitis (Lal et al., 2017), however both studies used the GreenGenes 13_8 taxonomic database for classification. This database does not contain *Dolosigranulum*, a nasopharyngeal commensal, and will misclassify it as *Alloiococcus*. These studies may similarly have not been able to detect *Turicella* in the nasopharynx as the GreenGenes database will report it as *Corynebacterium*. Similarly, nasopharyngeal microbiome studies that aggregate taxa at broader levels will have missed these genera. In 16S rRNA studies that did not use GreenGenes, *Alloiococcus*, and *Turicella* were not reported in the nasopharynx of children with OM (Laufer et al., 2011; Pettigrew et al., 2012; Jervis-Bardy et al., 2015). *Alloiococcus* was present at very low relative abundance in nasopharyngeal samples from children with rAOM (0.19% across all samples) and in healthy controls (0.17%) in our own study—significantly lower abundance than in the MEF (49.8%) (Lappan et al., 2018). *Turicella* was similarly present at 0.03% in the nasopharynx of both groups of children, again significantly lower than in the MEF (6.7%) (Lappan et al., 2018). In the nasopharynx, both genera were present at a similar level to known contaminants in negative control samples, indicating that caution should be taken to interpret the organisms as present in the nasopharynx.

These studies indicate that if *A. otitidis* and *T. otitidis* are present in the nasopharynx of children with OM then it is only at low abundance; they appear to be even more scarce in the nasopharynx of healthy individuals. The nasopharynx is therefore unlikely to represent a reservoir for these organisms, or their presence there is transient and potentially originates from the middle ear. If they do not originate from the nasopharynx, then it is also unlikely that they follow the same pathway as the major otopathogens to the middle ear. This may be because these organisms are indeed not otopathogens, or it may be because the EAC, rather than the nasopharynx, is their predominant niche. The studies summarized so far do not provide any causal or mechanistic link between the presence of these organisms and the development of OM.

## Are These Organisms Capable of Causing Disease?

In addition to understanding their mechanism for colonizing the middle ear (and indeed confirmation that they are not simply contaminants from the EAC introduced during sampling), the other aspect of *A. otitidis* and *T. otitidis'* ecology that we lack is an understanding of whether they are capable of causing disease. Very few studies have attempted to investigate the behavior and potential pathogenic activity of these two species, with all published articles that have investigated this issue focusing on *A. otitidis*. Evaluating their pathogenic potential is an integral part of understanding whether these organisms are causative of OM, if they contribute indirectly to pathogenesis, or whether they are aural commensals that are not involved in the pathogenesis of OM.

### *A. otitidis* Provokes an Immune Response

There are a handful of studies that have assessed the ability of *A. otitidis* to elicit an immune response, as a proxy measure to indicate pathogenic potential. Initial studies assessed the production of interleukins (IL) from THP-1 cells (IL-8 and IL-12) and Hep-2, HeLa and U937 cells (IL-8) in response to *A. otitidis*. These studies demonstrated that viable *A. otitidis* was capable of stimulating IL-8 and IL-12 at similar levels to the three major otopathogens (Himi et al., 2000; Kita et al., 2000). The effect was reduced when cells were stimulated with whole killed *A. otitidis* and when viable cells were prevented from direct contact, but soluble proteins from *A. otitidis* elicited a response for both cytokines (Himi et al., 2000; Kita et al., 2000). Whole killed *A. otitidis* can also induce expression of CD69 (indicating activation of lymphocytes) in blood and adenoidal lymphocytes at comparable levels to the major otopathogens (Harimaya et al., 2005), but the response is lower than that induced by *Staphylococcus aureus* (Tarkkanen et al., 2000). Whole killed *A. otitidis* has also been shown to activate signaling pathways for the production of IL-8, similarly to *S. pneumoniae* (Harimaya et al., 2007a). Formalin-killed cells from clinical isolates of *A. otitidis* have induced IL-1β, IL-6, IL-8, and TNF-α on a cell line *in vitro* at or above levels induced by *S. pneumoniae* (Ashhurst-Smith et al., 2014a). An experiment with cell-free filtrates from these isolates indicated that extracellular proteins rather than peptodiglycan may be responsible for the induction (Ashhurst-Smith et al., 2014b).

The production of pro-inflammatory cytokines provides preliminary evidence that the human immune system responds to *A. otitidis*. However, these studies lack replication in a middle ear epithelial environment, have not involved comparisons with non-pathogenic bacteria, and are potentially strain-dependent. For example, some strains of the nasopharyngeal commensal *Haemophilus haemolyticus* have been shown to elicit an inflammatory response *in vitro* (Pickering et al., 2016). It remains unclear from these studies whether these inflammatory responses are characteristic of the response in the middle ear of children with OM, and whether they are evidence for pathogenicity.

Two further studies by Harimaya et al. have explored the immune response to *A. otitidis* within the middle ear. In 2007, they determined that MEF from children with *A. otitidis-*positive OM (in the absence of other detectable otopathogens) contained IgG, secretory IgA, IgG2, and IgM specific to *A. otitidis* (Harimaya et al., 2007b). In 2009, they also discovered that *A. otitidis*-positive MEF contained similar levels of pro-inflammatory cytokines and chemokines as *S. pneumoniae*-positive MEF (both in the absence of other otopathogens detectable by culture or PCR) (Harimaya et al., 2009). While these preliminary studies require replication, it is important to note that *A. otitidis* does appear to elicit an innate and adaptive immune response in the middle ear of children with OM.

### Observations of Bacterial Activity

There is also some limited evidence for *Alloiococcus* having pathogenic potential based on how the organism behaves. Upon its initial discovery, *Alloiococcus* was reported to be found intracellularly, which the authors suggested was an indication of pathogenic capability (Faden and Dryja, 1989); though this may have been an observation of engulfment by neutrophils. Two reports of intracellular bacteria found in the middle ear mucosa described Gram positive cocci (Coates et al., 2008) and other, unidentified bacteria (Thornton et al., 2011), though no studies since have indicated the presence of intracellular *Alloiococcus*. In children with AOM and perforation, the *Alloiococcus* bacterial load in MEF was comparable to *H. influenzae* (Marsh et al., 2012). *A. otitidis* has been observed more frequently than the major otopathogens in non-purulent MEF (Holder et al., 2012, 2015) and more commonly in persistent (≥3 month duration) effusions than those of shorter duration (Leskinen et al., 2002), suggesting involvement in chronicity. It is similarly prevalent in purulent effusions (Holder et al., 2012, 2015), though its presence in the MEF of children with AOM appears to be unrelated to disease severity (Leskinen et al., 2004). *A. otitidis* is strictly aerobic, as its viability decreases greatly when grown anaerobically (Matsuo et al., 2011). Its presence in middle ear fluids is therefore somewhat unexpected, as the fluid-filled middle ear in OM is thought to be hypoxic (Cheeseman et al., 2011). Interestingly, the organism has recently been shown to form biofilms in broth culture, both with *H. influenzae* and independently as a single-species biofilm (Chan et al., 2017a). Furthermore, *A. otitidis* improved the growth of *H. influenzae* when in media without its required X and V factors and at suboptimal temperatures, suggesting its enhancement of biofilm production by otopathogens may allow *A. otitidis* to indirectly contribute to the pathogenesis of OM. This multispecies biofilm was also less susceptible to antimicrobial killing, and it is possible that *A. otitidis*' ability to also form biofilm alone may contribute to the perpetuation of inflammation and chronic OM (Chan et al., 2017a). Understanding whether *A. otitidis* DNA detected in MEF originates from live or dead cells, and whether residence in biofilm improves its tolerance to low oxygen conditions would be highly informative regarding its persistence in the middle ear.

For *Turicella*, very little has been investigated with respect to pathogenicity other than its resistance to antimicrobials. *Turicella* strains have shown resistance to macrolides and clindamycin (Gómez-Garcés et al., 2007; Boumghar-Bourtchai et al., 2009), though are susceptible to amoxicillin-clavulanic acid (Funke et al., 1996; Troxler et al., 2001) which is more commonly used to treat AOM (Antibiotic Expert Group, 2014). The draft genome sequence of a strain isolated from MEF of a child with OM apparently did not contain any candidate virulence factors, but no detail was given as to how these were sought (Brinkrolf et al., 2012). We therefore currently have minimal evidence for the pathogenicity of *T. otitidis*.

### Assessment in Animal Models

To our knowledge, there has only been one published study investigating *Alloiococcus* in an animal model to evaluate pathogenicity, and none investigating *Turicella*. In 2008, Tano et al. inoculated >10^8^ CFU/ml of *Alloiococcus* into the middle ear of 7 rats (a 10-fold higher concentration than required of the major otopathogens) (Tano et al., 2008). Reactions were mild; after 3 days, all rats had an amber-colored (but not purulent) effusion in the middle ear but by day 14 all ears were normal. In conjunction with their observations that *Alloiococcus* was found in the EAC of healthy adults and rarely in the nasopharynx of children, Tano et al. concluded that *Alloiococcus* is not an otopathogen. This study requires replication, though it suggests that *A. otitidis* is not overtly pathogenic on its own; an observation consistent with the association of *A. otitidis* with OME and persistent inflammation rather than AOM. However, effective animal models of AOM often make use of a viral infection to predispose the animal to AOM, as occurs in humans (Davidoss et al., 2018); so it remains possible that *A. otitidis* would produce or exacerbate disease in the presence of a co-infecting virus or organism. This would be congruent with the observations of coinfection summarized in Table 2, and with *A. otitidis'* ability to enhance the survival of NTHi in biofilm (Chan et al., 2017a).

### Pathogenicity in Diseases Other Than Otitis Media

There have been isolated reports of *A. otitidis* and *T. otitidis* as causative agents of diseases seemingly unrelated to otitis media. *A. otitidis* has been described as the causative agent in two case reports, where it was isolated from vitreous fluid of a patient with acute-onset endopthalmitis (Marchino et al., 2013) and from blood cultures in a young adult with endocarditis (Guler et al., 2015). Both infections are usually caused by the Gram positive cocci *Streptococcus* and *Staphylococcus* (Marchino et al., 2013; Guler et al., 2015); it is possible that the cause was misidentified as these reports do not provide any detail on how the isolates were identified as *A. otitidis*. In the endocarditis case, the patient had chronic OME with perforation which had apparently been a problem since childhood, so it is possible that in long-term severe disease *A. otitidis* can opportunistically infect the bloodstream. *T. otitidis* has similarly had few instances of unrelated opportunistic infection. Two case reports isolated *T. otitidis* from the blood of an immunocompromised child (Loïez et al., 2002) and an elderly hospitalized patient (Bîrlutiu et al., 2017) with bacteraemia. In the case of the child, otitis externa was apparently present at the time and *T. otitidis* was also isolated from an external ear swab (Loïez et al., 2002). It has also been isolated in pure culture from a posterior auricular abscess (Reynolds et al., 2001) and a case of mastoiditis (Dana et al., 2001), both in young children. The *T. otitidis* strain TD1, whose genome was recently sequenced (Greninger et al., 2015), was isolated from a venous catheter. It is therefore plausible that the genomic contents of this strain are not representative of strains involved in OM.

Thus, there is weak evidence for both *A. otitidis* and *T. otitidis* to have opportunistic pathogenicity outside of the ear; these reports are infrequent and not replicated. Samples taken from in or around the ear have the potential to have been contaminated with the organisms from the EAC; outside of these few case reports these species have only been reported in the middle or external ear. It is unknown how these organisms would migrate to the blood, and it is likely that the connection between these patients' ear disease and bacteraemia is a result of sample contamination or transient bacteraemia rather than invasive infection by these organisms.

These sparse reports of pathogenic activity are in contrast to the major otopathogens, each of which can reside asymptomatically but are also responsible for a wide range of infections (Verduin et al., 2002; Bogaert et al., 2004; Van Eldere et al., 2014). Overall, there is currently minimal evidence for the pathogenicity of *A. otitidis* and *T. otitidis* as the immunological and animal studies lack a comparison to commensal organisms known *not* to cause OM, and the work is lacking replication.

## Summary and Future Directions

### What we Currently Know

Our summary of the literature available at the time of writing suggests that *A. otitidis* and *T. otitidis* are primarily organisms of the human ear. They are found abundantly in both the MEF and EAC of children with OM, but are also present in the EAC of healthy children and adults. Current evidence suggests that *A. otitidis* and *T. otitidis* in MEF may originate from the EAC as they are uncommonly found in the nasopharynx. This pattern of detection is opposite to what is commonly observed of the major otopathogens, which typically colonize the nasopharynx to ascend to the middle ear and are rarely found in the EAC. Curiously, the closest relatives of *Alloiococcus* and *Turicella* are *Dolosigranulum*, and *Corynebacterium*, respectively; both organisms being usual nasopharyngeal commensals. These organisms may have diverged to inhabit the middle ear and the nasopharynx, where they have potentially developed specialized but very different interactions with the other dominant organisms in these niches (the otopathogen genera).

The majority of available studies on *A. otitidis* and *T. otitidis* only *hypothesize* their role in the pathogenesis of OM by describing their prevalence and abundance, but relatively few studies set out to *test* these hypotheses to better define their role in disease. Thus, we have minimal information on whether or how these organisms contribute to the development of OM.

The few studies that have aimed to directly investigate pathogenic potential have tested only *A. otitidis*. There is evidence that it is capable of forming biofilm and supporting the survival of NTHi, stimulating release of pro-inflammatory cytokines *in vitro*, and stimulating the production of specific antibodies. However, these studies have compared *A. otitidis* only with the major otopathogens and not with known commensal species; it is possible that a commensal organism would still elicit these responses. There is still much that is unknown about the role of *A. otitidis* and *T. otitidis* in the pathogenesis of OM, and a targeted research effort is required to further characterize their function.

### Designing Future Studies to Clarify Their Role in Otitis Media

Even after more than 25 years since their discovery, there is much about the behavior of *A. otitidis* and *T. otitidis* in the middle ear and EAC that remains unknown. Firstly, while there are many studies describing their prevalence in the MEF of children with OM, and some in the EAC, these studies are often confounded with the possibility of EAC flora contaminating MEF samples during collection. However, several studies have reported the presence of *A. otitidis* or *T. otitidis* in specimens from children in which tympanic membrane perforations (including previous grommets) had not occurred (Hendolin et al., 1999; Leskinen et al., 2002, 2004; de Miguel Martínez and Ramos Macías, 2008; Khoramrooz et al., 2012). This suggests three possibilities: 1) *A. otitidis* and *T. otitidis* inhabit the normal middle ear, prior to the onset of OM; 2) they enter the middle ear through undetectable, minor tympanic membrane perforations; 3) they are detectable in MEF specimens as a result of contamination from the EAC during sampling.

The first possibility is challenging to address, as the healthy middle ear is a very low biomass environment, where samples are highly prone to environmental contamination and the area is inaccessible without surgery. Some attempts have been made to survey the microbiota of the healthy middle ear, and these studies have not consistently reported the presence of *A. otitidis* and *T. otitidis* (Antonelli and Ojano-Dirain, 2013; Neeff et al., 2016; Minami et al., 2017) and some have not found evidence for the presence of bacteria in the normal middle ear at all (de Miguel Martínez and Ramos Macías, 2008; Westerberg et al., 2009; Thornton et al., 2011; Papp et al., 2016). The second possibility is also challenging, but may be explored with animal or tissue models of an inflamed tympanic membrane to test its permeability to bacteria. These will also be important studies if it is found that viable *A. otitidis* and *T. otitidis* in the MEF originate from the EAC, even in children with intact tympanic membranes. The final possibility of MEF contamination is the most straightforward to test, and these experiments are also useful for addressing the second possibility. A well-designed study aiming to isolate viable *A. otitidis* and *T. otitidis* from the two sites independently is required. This may be achieved by sampling the EAC, sterilizing it thoroughly, sampling it again, and then opening the tympanic membrane to sample the MEF with all care taken not to contact the EAC. This should be carried out in children who have never had a known tympanic membrane perforation as well as those who have, or have had tympanostomy tubes in the past. Such a study would also be well-positioned to answer the subsequent question, addressing the minor perforation hypothesis: if these organisms reside in the MEF, how do they get there? The isolates from the EAC and MEF of the same ear may be phenotypically or genetically compared to determine if the EAC is the likely origin of the isolates from the MEF. Higher similarity between EAC and MEF strains of the same patient than between strains from different patients may indicate the transferral of strains between the EAC and MEF in the absence of sampling contamination. Metagenomics is also a useful tool for answering this question if isolation of live bacteria is a challenge (which could be informative in itself). Metagenomic analysis would also allow for the characterization of other EAC community members to aid in the assessment of contamination and strain relatedness between the EAC and MEF communities. Bacterial load estimation via species-specific qPCR would also be useful to determine whether there is a larger population of *A. otitidis* and *T. otitidis* in the EAC or in the MEF, keeping in mind that there may be differences in the live and dead populations of *A. otitidis* and *T. otitidis*. Longitudinal sampling of the EAC may also indicate whether the presence or abundance of these organisms in the EAC is associated with episodes of OM. These experiments should provide a solid body of evidence to a) show whether *A. otitidis* and *T. otitidis* do inhabit the MEF and are not present due to contamination; and b) whether they enter the middle ear from the EAC, and how they do so.

The second major gap in current knowledge is that the functions these organisms perform in the EAC and MEF is entirely unknown. To begin to understand the behavior of these organisms in the middle ear, metagenomics and other new “'omics” techniques may be able to answer many of these questions. The genomes of *A. otitidis* and *T. otitidis* isolates are currently in draft stage; complete, annotated genomes will allow us to understand whether they contain genes that may allow them to cause disease. The assembly of many additional genomes will aid an understanding of their metabolic functions and strain heterogeneity Transcriptomics and proteomics analysis of MEF could directly indicate their activity in the inflamed middle ear; this could be compared to their activity in the EAC. *In vitro* analyses making use of middle ear epithelial cell lines could confirm the pro-inflammatory stimulation by *A. otitidis*, and *T. otitidis* could be studied in this manner as well. These experiments would also be useful to determine whether the species are capable of residing intracellularly in the middle ear mucosa, as a mechanism for immune or antibiotic evasion. Further work with animal models would also fill gaps in our knowledge of the behavior of *A. otitidis* and *T. otitidis* in the middle ear, perhaps allowing us to determine if the presence of these organisms in OM caused by the major otopathogens results in a more severe phenotype. With these models, we may determine whether these organisms can produce OM on their own, or whether the presence of otopathogens or viruses is required. It can be assessed whether the phenotype is more severe in the presence of *A. otitidis* or *T. otitidis*. Understanding what functions these organisms perform in the MEF is essential to understand whether they play a pathogenic role, by themselves or in synergism with the otopathgoens.

## Conclusion

Research focusing on the development of new therapies to treat severe OM has focused on the major otopathogens; with advances in the areas of vaccinations, tackling biofilm and restoring the commensal flora of the nasopharynx. Despite these advances in prevention and treatment, OM remains a very common childhood disease that can be difficult to treat. The complex polymicrobial nature of the disease and the role dominant organisms like *A. otitidis* and *T. otitidis* may play has remained equivocal for decades. The current understanding of these organisms is limited, and there are many ways in which they can be further characterized. It is important to understand their role, if any, in the pathogenesis of OM, as it is plausible that they are supporting otopathogen growth or contributing to the persistence and recurrence of chronic OM; potentially entering the middle ear through minor tympanic membrane perforations. These organisms may be useful targets for treatment, which are perpetually overlooked as their commensal residence in the external ear canal continues to confound efforts to understand their pathogenicity. Establishing their role in the otitis-prone middle ear will allow the advancement of new therapies, or will ensure that resources are not overspent investigating commensals that are uninvolved in the disease.

## Author Contributions

RL undertook the literature review and drafted the manuscript. SJ and CP provided critical feedback on the manuscript. All authors have read and approved the final manuscript.

### Conflict of Interest

The authors declare that the research was conducted in the absence of any commercial or financial relationships that could be construed as a potential conflict of interest.
